# The Skin Microbiome: Is It Affected by UV-induced Immune Suppression?

**DOI:** 10.3389/fmicb.2016.01235

**Published:** 2016-08-10

**Authors:** VijayKumar Patra, Scott N. Byrne, Peter Wolf

**Affiliations:** ^1^Research Unit for Photodermatology, Department of Dermatology, Medical University of GrazGraz, Austria; ^2^Center for Medical Research, Medical University of GrazGraz, Austria; ^3^Cellular Photoimmunology Group, Infectious Diseases and Immunology, Sydney Medical School, The Charles Perkins Center Hub at The University of Sydney, SydneyNSW, Australia

**Keywords:** skin microbiome, ultraviolet radiation, immune suppression, innate immunity, environmental factors

## Abstract

Human skin apart from functioning as a physical barricade to stop the entry of pathogens, also hosts innumerable commensal organisms. The skin cells and the immune system constantly interact with microbes, to maintain cutaneous homeostasis, despite the challenges offered by various environmental factors. A major environmental factor affecting the skin is ultraviolet radiation (UV-R) from sunlight. UV-R is well known to modulate the immune system, which can be both beneficial and deleterious. By targeting the cells and molecules within skin, UV-R can trigger the production and release of antimicrobial peptides, affect the innate immune system and ultimately suppress the adaptive cellular immune response. This can contribute to skin carcinogenesis and the promotion of infectious agents such as herpes simplex virus and possibly others. On the other hand, a UV-established immunosuppressive environment may protect against the induction of immunologically mediated skin diseases including some of photodermatoses such as polymorphic light eruption. In this article, we share our perspective about the possibility that UV-induced immune suppression may alter the landscape of the skin’s microbiome and its components. Alternatively, or in concert with this, direct UV-induced DNA and membrane damage to the microbiome may result in pathogen associated molecular patterns (PAMPs) that interfere with UV-induced immune suppression.

## Skin Microbiome

### Introduction

The human skin is the largest organ of the body with a diverse physical, chemical, and biological ecosystem over a surface of 1.8 m^2^. The epithelial surface engages itself in a mutualistic relationship with a wide range of composite microorganisms, including bacteria, fungi, viruses, and mites, all of them residing on or within the skin. Around 1 million bacteria reside per square centimeter of skin surface, making up an estimated total of 10^10^ bacterial cells growing on the entire skin of the human body (Grice et al., 2008; Hannigan and Grice, 2013). The majority of those bacteria are commensals or transients. The diversity of the skin’s microbiome is due to the diverse environments present on the skin, resulting from a divergent physical nature of the skin, humidity, temperature, pH, lipid and sebum content, as well as antimicrobial peptide (AMP) expression (Nakatsuji et al., 2013). Considering the capacity for a robust cutaneous immune system to rapidly detect and eliminate foreign invaders, it is intriguing that innumerable numbers of microorganisms reside on the skin surface and also extend to sub-epidermal compartments, associated appendages such as, hair follicles and sebaceous glands (Nakatsuji et al., 2013; Belkaid and Segre, 2014).

### Bacteria

Sequencing the bacterial 16S small-subunit ribosomal RNA gene reveals that the skin surface is dominated by *Proteobacteria, Actinobacteria, Bacteriodetes*, and *Firmicutes* (Grice et al., 2008). Colonization varies topographically; for example, *Proteobacteria* and *Staphylococcus* spp. are abundantly present on the skin surface and are deeply intertwined between themselves and other microorganisms. Recent studies indicate that specific bacterial communities are associated with moist, dry or sebaceous microenvironments of the skin. Moist areas such as umbilicus, the axillary vault, inguinal crease, gluteal crease, foot, popliteal fossa, and the antecubital fossa have an abundance of *Staphylococcus* and *Corynebacterium* spp. and these organisms are well known to prefer skin sites with high humidity. The dry areas such as buttocks, forearms and certain other parts of the hand have the most intertwined collection of phyla *Actinobacteria, Proteobacteria, Firmicutes*, and *Bacteriodetes* (Grice et al., 2008). Interestingly, these dry sites are known to have an abundance of gram-negative bacteria which were thought to colonize very rarely on the skin. When compared to the gut or the oral cavity, dry skin sites are home to a much greater phylogenetic diversity of bacteria (Gao et al., 2007; Costello et al., 2009; Grice et al., 2009). The lowest bacterial diversity is seen around the sebaceous sites, which suggests that only few bacterial communities can flourish under those conditions (Costello et al., 2009). The forehead, retroauricular crease (behind the ears), the back and the alar crease (sides of the nose) are few of the sebaceous sites containing low phylotype richness. *Propionibacterium* spp. dominate the sebaceous skin areas like the hair follicle, hair shaft, and the sebaceous gland (Costello et al., 2009).

### Fungi

The skin microbiome is not only limited to bacteria, but extends to fungal species as well, which have major roles in health, and disease. Genomic methods to characterize fungal species are very limited when compared to that available for the bacteria. Studies done by Findley et al. (2013) described fungal communities residing in the skin by sequencing 18s rRNA (phylogenetic marker within ribosomal RNA region) and ITS1 (internal transcribed spacer 1 region). They observed that the genus *Malassezia* predominated across the 11 core body and arm sites of their study (back, occiput, external auditory canal, inguinal crease, retroauricular crease, glabella, manubrium, nare, anticubital fossa, volar forearm, hypothenar palm, plantar heel, toe nail, and toe-web space). Plantar heel had the highest diversity of fungal species, with a mixed representation of *Malassezia, Aspergillus, Cryptococcus, Rhodotorula, Epicoccum*, and few others (Findley et al., 2013). ITS1 sequencing also revealed that *Candida* species like *tropicalis, parapsilosis*, and *orthopsilosis*, and *Cryptococcus* species *flavus, dimennae*, and *diﬄuens* were observed on and within the skin. This may be important as these species are thought to be potential pathogens in wounds of immunocompetent subjects or immunocompromised patients in general (Larone, 2002; Findley et al., 2013).

### Archaea

Probst et al. (2013) reported about the less known archaea found on the human skin based on the 16S rRNA sequencing. They observed around 4% of the overall microbial genes was found to be archaeal 16s rRNA genes. They also found that around 88% of all the observed operational taxonomical units (OTUs) consisted of phyla Thaumarchaeota with the rest being Euryarchaeota (Probst et al., 2013). As bacteria constitute the bulk of the microbiome, archaea have received little research attention. Advances in detection technologies now allow to detect all the archaeal taxa and study its potential prevalence on the skin and understand its impact on health and diseases. The prevalence of archaea within the human microbiome has been addressed in a recent review by Horz (2015).

### Viruses

Skin viral microbiota is one of the most rarely investigated subsets of the human microbiome, as the skin related viruses cannot be cultivated and do not portray consensus sequences to be detected by molecular methods. In recent years, there has been increasing evidence suggesting that healthy skin harbors resident or short-lived viruses, such as, human alpha, beta, and gamma papillomaviruses (α-HPV, β-HPV, and γ-HPV) present on and within the upper layers of human skin (Chen et al., 2008; Antonsson et al., 2003a,b). By applying the functional metagenomic methods (Hannigan et al., 2015) to skin samples have also led to observing new viral species such as *Polyomoaviridae* family (Foulongne et al., 2012). Foulongne et al. (2012) reported that eukaryotic DNA viruses on human skin consisted of *Papillomaviridae, Polyomoaviridae*, and *Circoviridae*. They also observed 13 new γ-HPV strains present on the healthy skin (Foulongne et al., 2012). These reports indicate the presence of cutaneous viral microbiota and their possible involvement in various proliferative skin diseases.

### Mites

Small arthropods such as *Demodex* mites (*Demodex folliculorum* and *Demodex brevis*) have long been associated with rosacea and other skin conditions such as chronic blepharitis (Georgala et al., 2001; Lacey et al., 2007; Elston, 2010). These mites are usually found on the facial skin, around the pilosebaceous glands and the hair follicles and are thought to be a part of commensal skin microbiota, as they have prevalence rates between 23 and 100% in healthy individuals (Norn, 1971; Rufli and Mumcuoglu, 1981). Furthermore, *Demodex mites* are also associated with cutaneous conditions of rosacea-like appearance, commonly clubbed as demodicosis or demodicidosis, though their presence on the skin most commonly remains asymptomatic.

### Factors Affecting Skin

The skin is one the most exposed organs of our body, which provides a physical defense from the external environment and most often reacts appropriately to the wide range of hazards encountered. To list only a few of the environmental hazards to the skin microbiome, exposure to UV-R, pollutants, climatic elements at certain geographic locations, occupation, and exaggerated hygiene have to be noted. Despite all these environmental assaults, the microbiome and the host must maintain cutaneous homeostasis to sustain normal physiological behavior.

## UV-R and Immune Suppression

**FIGURE 1:**
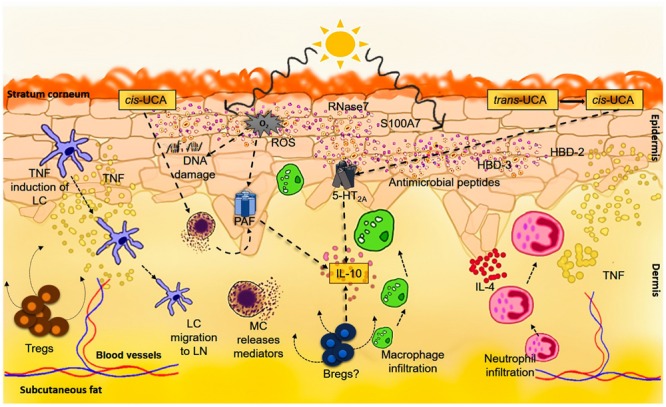
**Ultraviolet-induced immune suppression.** Exposure to UV-R results in DNA damage, *cis*-UCA formation as well as the production and release of platelet activating factor (PAF) and PAF-like molecules. Photoproducts of DNA such as pyrimidine dimers or 6-4-photoproducts result in the production and release of various immunosuppressive factors like TNF-alpha and IL-10 by keratinocytes and other cells in the skin. The UVB waveband in particular directly also leads to the isomerization of *trans*-UCA to *cis*-UCA. *Cis*-UCA induces immune suppression by binding to the 5-HT_2A_ receptor, in turn leading to production of IL-10 by T-cells and B-cells. It may also indirectly lead to mast cell degranulation and stimulate the release of PAF. Formation of reactive oxygen species (ROS) by UV-R not only induces and contributes to DNA damage but also directly stimulates PAF synthesis or the production of PAF-like molecules. UVR can also directly upregulate certain AMPs such as human beta-defensin (HBD)-2, -3, S100A7, and RNase7 which are expressed by keratinocytes, lymphocytes, monocytes, and mast cells. These AMPs not only serve as initiators of the innate immune response but they also communicate with and can activate the adaptive immune system. Together, the impact of UV-R on the skin induces an immunosuppressive microenvironment with abundance of TNF, IL-4 and IL-10 linked to Langerhans cell (LC) migration into the lymph nodes and neutrophil and macrophage recruitment to the skin. As overall result, there is induction of regulatory T cells (Tregs) and regulatory B cells (Bregs), leading ultimately to functional immune suppression.

Among the above mentioned factors, UV-R is one of the most prominent factors linked to skin hazards. UV-R, especially UV-B (280–315 nm) and UV-A (315–400 nm) are known to be involved in skin freckling, wrinkling, photo allergic, and phototoxic responses and tumor induction and progression (Krutmann, 2000; Lee et al., 2013). One of the widely known aspects of UV-R on the skin is the ability to induce photoproducts such as cyclobutane pyrimidine dimers and subsequently occurring mutations, which are linked to carcinogenesis (Lee et al., 2013). Immune suppression is also induced by UV-R and is considered to be another of its harmful impacts (Phan et al., 2006). Kripke et al. (1977) first discovered that UV-R exposure and immune suppression were linked to UV-induced carcinogenesis (Schwarz, 2010). Since then, the immunomodulating properties of UV-R were confirmed by employing contact hypersensitivity (CHS) models in mice (Toews et al., 1980; Elmets et al., 1983) as well as in humans (Cooper et al., 1992; Kelly et al., 2000; Wolf et al., 2003). UV-R-induced immune suppression is known be mediated through T cells (Elmets et al., 1983). The relation of immune suppression linked to various subtypes of regulatory immune cells such as regulatory T cells (Tregs) (Schwarz, 2008; Schweintzger et al., 2015; Schweintzger et al., 2016) and regulatory B cells (Bregs) (Byrne and Halliday, 2005) depend on UV-R doses, antigens and type of immune response (i.e., CHS vs. DTH). Some of the key events observed in the skin after the UV-R exposure are shown in **Figure 1**. The most studied photochemical reactions triggered by UV-R are DNA damage (Applegate et al., 1989), isomerization of urocanic acid (De Fabo and Noonan, 1983), and formation of reactive biophospholipids such as platelet activating factor (PAF) (Wolf et al., 2006) in the skin. Cytokines such as tumor necrosis factor (TNF)-α (Wolf et al., 2000), interleukin (IL)-4, IL-10, IL-33 (Byrne et al., 2011), and prostaglandin E_2_ (Shreedhar et al., 1998) are expressed and upregulated after UV-R exposure. In healthy skin, the expression of these cytokines by UV-R leads to infiltration of suppressor macrophage and neutrophils (Cooper et al., 1985, 1986, 1993. Furthermore, UV-R also induces the emigration of Langerhans cells (LC) from the epidermis into the draining lymph nodes (Toews et al., 1980; Noonan et al., 1984) and affects mast cells which are known to be involved in immune suppression (Hart et al., 2001). In addition to DNA damage, *cis*-urocanic acid (UCA) is a UV-induced immunosuppressive molecule (De Fabo and Noonan, 1983), acting via the 5-HT_2A_ receptor (Walterscheid et al., 2006; Wolf et al., 2016). Upon exposure to UV-R, *trans*-urocanic acid (*trans*-UCA) is converted to the *cis* isoform, which accumulates on the stratum corneum and epidermis. Kubica et al. (2014) observed temperate changes in the skin microbiome of capase-14 deficient mice, which are generally characterized by reduced overall levels of UCA (Kubica et al., 2014). Interestingly, it is known that caspase-14 controls the proteolysis of fillagrin and patients with fillagrin mutations are more likely to develop atopic dermatitis (AD), which in turn is associated with alterations of microbial load on the skin (McAleer and Irvine, 2013). Another immunosuppressive factor released upon UV exposure is PAF that binds to the PAF receptor, resulting in a cascade of downstream events that lead to the release of IL-10, and ultimately immune suppression (Walterscheid et al., 2002; Wolf et al., 2006).

Since UV-R suppresses the immune system and previous research has shown in experimental models that UV-R can suppress the immune response to infectious microorganisms (Chapman et al., 1995), one can speculate that exposure to UV-R could enhance susceptibility to microbial infections and/or it could worsen infectious diseases. However, clinical evidence of the increased infections after UV-R exposure remains very low, with a few exceptions. It has been known for a long time that UV exposure can trigger and/or exacerbate herpes simplex virus (HSV) manifestations (Norval, 2006). A UV dose dependent induction was observed on the lips of patients harboring the latent infections of HSV-1 (type 1). This was also reported in cases of HSV-2 (type 2). Local modifications of the immune system are thought to be the involved in the reactivation of the latent HSV infection (Wheeler, 1975; Tesini et al., 1977; Spruance, 1985). In immunocompetent humans, *Candida albicans* is known to cause (minor) infections of the skin and mucosa of the genital and gastrointestinal tract, however, in immunosuppressed patients it can cause life-threatening systemic infections. Importantly to note, suppression of the response to *candida albicans* antigen is used in one of the standard assays to quantify immune suppression induced by UV-R (Wolf et al., 2016). In mice, it is also known that UV-R decreases the delayed type hypersensitivity (DTH) responses and significantly modifies the course of the infection caused by *Mycobacterium bovis* bacillus Calmette- Guérin (BCG), which is closely related to the organism causing tuberculosis in humans (Jeevan and Kripke, 1989). Higher number of viable bacilli were observed in the peripheral lymph nodes of mice irradiated with UV, compared to that of unirradiated mice (Jeevan and Kripke, 1989). This phenomenon was similarly observed in mice infected with *Mycobacterium lepraemurium*, a pathogen known to cause infections which to some extent resemble leprosy in humans (Jeevan et al., 1992). In humans, experimental exposure to UV-R reduced the granulomatous reaction in individuals sensitized with lepromin (antigens of *Mycobacterium lepraemurium*) (Cestari et al., 1995). Brown et al. (2001) reported that exposure to UV-R can alter the immune responses in mice infected by *Borrelia burgdorferi* and intensify the associated arthritic component (that resembles Lyme disease in humans) (Brown et al., 2001).

### Antimicrobial Peptides

AMPs are small proteins, known to have microbicidal activity. Besides being microbicidal, they possess immunomodulating properties. AMPs are mostly produced by the cells in constant exposure to microorganisms. In the skin there are two main classes of AMPs, i.e., β-defensins and cathelicidins. In humans LL-37 is the only cathelicidin found, which is expressed by various epithelial cells such as keratinocytes in inflamed skin (Niyonsaba et al., 2007). Apart from defensins and cathelicidins, skin also constitutively expresses a wide range of AMPs like RNase 7 (ribonuclease 7), S100A7 (Psoriasin, calcium binding protein), and dermcidin (that is sweat gland derived). AMPs production can be increased during inflammation or in cases of infection. The production of these AMPs, including beta defensins, cathelicidins, ribonucleases, and S100 proteins, are triggered by various pathogen or damage associated molecular patterns (PAMPs/DAMPs), exogenous microbial danger signals like Toll-like receptor (TLR) agonists, or endogenous mediators of inflammation such as TNF-α, IL-1, IFN-γ, and IL-17 (Biragyn et al., 2002; Chadebech et al., 2003; Joly et al., 2005; Kolls et al., 2008). It is also known that UV-R induces productio of AMPs, as essential components and triggers of the innate immune system. Studies have shown that UV-R induces human beta defensin 2 (hBD2), hBD3, ribonuclease 7 (RNase7), S100A7 (psoriasin), S100A12 and elafin by keratinocytes *in vitro* and *in vivo* (Yang et al., 2002; Hong et al., 2008; Gläser et al., 2009; Felton et al., 2013; Kennedy Crispin et al., 2013). One mechanism of UV-induction of AMPs could involve production of the active form of Vitamin D (Wang et al., 2004). Alternatively, or in concert with this, Vitamin D_3_ itself could be suppressing adaptive immune responses (Damian et al., 2010) and/or tempering inflammatory events in UV-exposed skin (Mason et al., 2010).

Apart from participating in innate immune responses, AMPs are also involved in activating and mediating adaptive immune responses (Yang et al., 1999, 2002; Biragyn et al., 2002; Niyonsaba et al., 2007; Navid et al., 2012). The much abundant skin’s physiologically beneficial microbiome vastly depends upon AMPs to be kept and maintained in homeostasis in order to release and allow the immune system to mount an immune response, when needed, and protecting against invading pathogens.

## UV-R, Skin Microbiome, and Immune System Interaction

**FIGURE 2:**
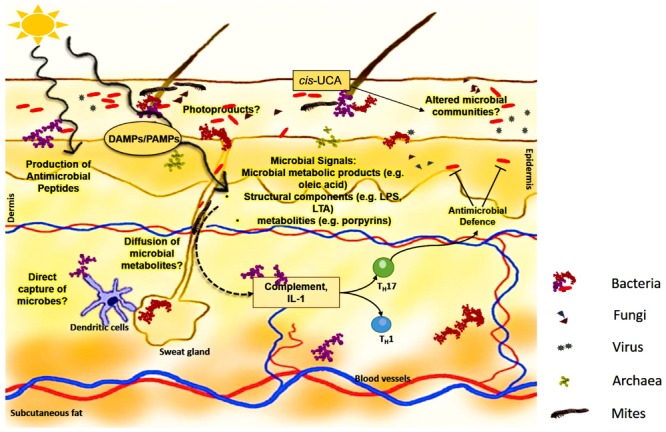
**Potential mechanisms of microbial interference in UV-induced immune suppression.** Microbiome, consisting of bacteria, fungi, viruses, archaea, and mites, covers the skin surface as well as colonizes in appendages and glands. This cutaneous microbiome encounters similar UV-R as skin cells do. UV-R could directly alter the microbial communities on the skin, by producing microbial photoproducts such as pyrimidine dimers and/or 6-4 photoproducts, leading to microbial killing. At sub toxic levels, UVR may initiate a pathogen/damage-associated molecular pattern (PAMP/DAMP) response. Such a response may result in the expression of various microbial signals such as oleic acid, LPS and/or porphyrins, affecting the overall immune signaling cascade, leading to inflammation, and altered immune response. Microbial metabolites can also exert an effect on dendritic cells which can recognize or can be involved in direct capture of microbes. Moreover, microorganisms can produce natural AMPs directly or can control AMP production by keratinocytes and their production can be increased by exposure to UV-R. UVR-induced *cis*-UCA could not only contribute to induction of altered immune response but also indirectly change the microbial load, by affecting the microenvironment through yet unknown pathways. In addition, the microbiome can also induce complement and IL-1 set under stress by UV-R and together with directly induced microbial signals influence the skin immunity by induction of various cytokines such as that of Th17 pathway. Said so, keratinocyte effector function could be influenced by production of IL-17, leading in circle to altered AMP production, and release, in turn affecting the microbiome.

In the last years the cellular components of the acquired (i.e., adaptive) immune response and to a lesser extent of the innate immune system and its changes after UV-R were characterized extensively. To date, relatively little is known about the effect of UV-R on the microbiome of the skin and how this affects the immune response after UV-R. Since the skin microbiome is established all over the skin surface and reaches deep down into appendages, logic dictates that it experiences similar impacts from UV-R as mammalian skin cells do (**Figure 2**). This exposure to UV-R can alter/damage the microbial community, possibly resulting in disruption of microbial components and/or formation of bacterial antigens, some of which may become immunogenic.

### UV-R and the Skin Microbiome

Much of the skin microbiome at sun-exposed body sites is directly exposed to solar UV-R, either completely or during much of its life cycle. UV-R can impose an intense change in the microbial species and its genotypic composition at exposed sites, depending upon the exposure time and intensity of UV-R. Some bacteria and fungi show a selective tolerance to UV-R for part of their life cycles, and are often vulnerable to the effects of UV during sporulation, diffusion and other processes such as infection. One of the major effects of UV-R on microbes is DNA damage, which can result in an increase in genetic variation or can alter the landscape of the microbial communities, thus disrupting the healthy microbiome (Rothschild, 1999). However, not all microbes are susceptible to the damaging effects of UV-R. For instance, many fungi show photomorphogenic effects upon exposure to UV-R. Wang et al. (2012) showed that the production of porphyrins by *Propionibacterium acnes (P. acnes)* was decreased with increased doses of UV-R, which provides evidence that facial bacteria are responsive to UV-R. They also observed the decrease of porphyrin production by *P. acnes*, at doses lower than 20 mJ/cm^2^ of UV-B. This indicates that *P. acnes* responds to UV-B even before a significant skin injury can be detected (Wang et al., 2012).

*Malassezia* spp. which also belong to the commensal microflora, is commonly causing pityriasis versicolor, a common skin disease condition particularly occurring in tropical regions but also moderate latitudes. At the site of the typical brownish-white scaly skin lesions of manifested disease, a sunburn response can hardly be provoked. Pityriacitrin, a UV-filtering compound which is produced by *Malassezia furfur* is believed to be protective. On the other hand, UV-R is well known to inhibit the cellular growth of *Malassezia furfur* (Wikler et al., 1990). Machowinski et al. (2006) looked into the effects of pityriacitrin on the other commensals residing on skin and observed that *Malassezia/Pityrosporum* was inhibited by UV-R and was much more sensitive than other commensals. It is hypothesized that fungi developed this UV-filter to reduce UV sensitivity which helps to grow and survive by competing with other commensals. However, Machowinski et al. (2006) they did not find any negative effect of pityriacitrin on other common skin commensals such as *Staphyloccoccus aureus, Staphylococcus epidermidis*, or *Candida albicans*.

Among viral populations, UV-R is known to be a stimulus for HSV. UV-R has been found to activate herpes virus promotor(s) and transcription factors such as c-jun (Stein et al., 1992; Loiacono et al., 2003). However, the complete mechanisms by which UV-R triggers clinical HSV manifestations have not been understood so far, but it is thought that UV effects on the immune system may contribute. In addition, there is some evidence that UV exposure may be a reactivation trigger of latent Varicella zoster virus infection, resulting in herpes zoster (Zak-Prelich et al., 2002).

Human papillomaviruses (HPVs), especially which come under the group epidermodysplasia verruciformis (EV) types, are known to be widespread on human skin (Norval, 2006). The EV HPV types have been linked to skin carcinogenesis in the genetic disease of EV (Lewandowsky-Lutz dysplasia). However, EV HPV types are also often found in non-melanoma skin cancers of the normal population though their pathologic significance in the later population remains uncertain. HPV appears to be part of the commensal flora of the skin, making it a challenge identifying causal relationships between HPV presence and skin conditions, including cancer (Neale et al., 2013). For instance, EV HPV was also found in hair follicles of psoriasis patients, in particular treated with psoralen + UV-A radiation, whereas its expression was not observed in patients who had received no treatment (Wolf et al., 2004). Together, however, there is increasing evidence suggesting that UV-R could affect the homeostasis between certain EV HPV types and the host, thus driving the infection from a latent stage to potentially oncogenic (Norval, 2006).

Patients suffering from AD were shown to have increased *Staphylococcus aureus* colonization on the skin, which was reduced after treatment with UV-B. Interestingly, UV-B had no effect on *Staphylococcus epidermidis*, possibly due to the fact that *Staphylococcus epidermidis* is mostly located around hair follicles, whereas *Staphylococcus aureus* is present on superficial skin layers more accessible for UV-B (Silva et al., 2006; Dotterud et al., 2008). Another study covered the effect of antimicrobial photodynamic therapy (APDT) which involved killing of microbes using light along with a photosensitizer. Almost all species used in the study were susceptible to APDT *in vitro*. Killing of *Staphylococcus epidermidis* and *Staphylococcus aureus* was significantly higher with natural sunlight than with polychromatic visible light produced by a standard slide projector (Zeina et al., 2001). Intriguingly, PDT was found to be effective in antibiotic-resistant folliculitis (Horn and Wolf, 2007). Blue light treatment (Charakida et al., 2004; Noborio et al., 2007) and conventional UV phototherapy (Rassai et al., 2014) may act beneficially in acne vulgaris by altering the skin microbiome and reducing *Propionibacterium acnes* density. Indeed, UV-R is known to be bactericidal and can break lipopolysaccharides (LPSs), lipoteichoic acids (LTAs) and other bacterial metabolites which have immunomodulatory properties (Liu et al., 2010; Weill et al., 2013). Moreover, PDT light treatment may not only work by direct effects on microorganisms but also by modulating the immune response directed against them (Reginato et al., 2014). However, the exact effects of UV-R on the skin microbiome are largely unexplored and its effects on archaea or skin mites is least known. In depth studies are warranted to understand the effects of UV-R, as with the emerging evidence of the wide arrangement of skin microbiome with the host immune system.

### Skin Microbiome-Immunity Dialog

The skin immune system and the microbiome have to be in constant communication in order to establish an equilibrium with each other. For this reason, it is important that the immune response is tailored to the appropriate threat, as any immune reaction toward commensals could lead to inflammatory responses and subsequent disease (SanMiguel and Grice, 2015). To execute these functions, skin is equipped with specialized immune cells of innate and adaptive immune system. An acute damage to skin will lead to production of certain ligands, which can activate keratinocytes and eventually results in the release of inflammatory mediators. Under these conditions LPA, a product of *Staphylococcus epidermidis* can reduce inflammation and take part in wound healing by binding to Toll-like receptor-2 (TLR-2), which is one of the innate immune receptors (Lai et al., 2009). Recently it has been reported that microbial LPS immunogenicity can lead to autoimmunity in humans (Vatanen et al., 2016). Skin microbiome has the capacity to control the expression of a wide range of innate immune sensors, such as AMPs (Gallo and Hooper, 2012). Most of the AMPs are known be constitutively expressed in the skin, and they can also be triggered by defined microbiota like *Propionibacterium* species and other gram-positive bacteria (Nagy et al., 2006; Lee et al., 2008; Gallo and Hooper, 2012). The mechanisms by which AMPs shape the microbial landscape remain to be clarified, as AMPs are induced by both UV-R and certain microbes. The multi-directional interaction between AMPs, microbes, and UV-R could be a key factor for the ecosystem of the skin microbiome, and could provide insight into the physiology of healthy skin as well as pathophysiology various acute and chronic inflammatory disorders.

The skin microbiome also induces the expression of other conserved pathways of the host immune system, such as components of the complement systems which contain large amounts of proteins. These proteins can react with each other and can take part in opsonization of a pathogen and induce other inflammatory responses to clear the pathogens (Belkaid and Segre, 2014). Germ-free mice, which are raised in absence of microbes, are known that they have a decreased expression of the C5aR, a component of the complement system, which results in reduced expression of AMPs and other pro-inflammatory factors. These are the changes which are commonly associated with dysbiosis of the skin microbiome (Naik et al., 2012; Chehoud et al., 2013). Exposure of mice to UV-R is known to activate both the classical (Hammerberg et al., 1998) and alternative (Stapelberg et al., 2009) complement pathways in skin, an event that precipitates immune suppression. However, the impact UV-activation of complement has on commensals remains to be investigated.

The skin microbiome itself can also control the expression of IL-1, which is actively involved in initiating and amplifying immune response (Naik et al., 2012). Upregulation of innate immunity by the skin microbiome in this way may lead to subsequent activation of the adaptive immune system. Indeed, the skin microbiome is known to functionally modulate T-cells by adjusting the local innate immune setting, especially via IL-1 production. This ultimately leads to increased production of pro-inflammatory cytokines such as IL-17A and interferon-γ (IFN- γ) by T cells.

Commensals may have evolved by distinctly controlling the network of the immune system depending upon environmental conditions. Since the skin has one of the largest pools of immune cells, and there is an enormous amount of pressure exerted by the skin microbiome, the cells of the innate immune system of the skin may commonly recognize the skin microbial antigens and prevent spread (Belkaid and Segre, 2014). Moreover, mice lacking adaptive immunity fail to recognize and restrain their commensal skin microbiota which leads to microbial diffusion to the local lymph nodes (Shen et al., 2014). In perspective with inflammation, various changes within the skin such as barrier permeability or increased contact with the resident microbiome can increase the local immune responses and with the ability of the skin microbiome to co-control both innate and adaptive immune response. Thus, the skin microbiome is likely the main driver and amplifier of various skin pathologies (**Figure 2**). Finally, DNA repair enzymes produced by evolutionary UV-resistant microbes such us *Micrococcus luteus* colonizing the human skin (Tomlin et al., 1978) may be capable of not only repairing UV-induced damage to their own DNA but potentially transfer this help to other commensals and possibly even cells of the human skin. For instance, endonuclease T4N5 from the bacteriophage T4 encapsulated in multilamellar liposomes can penetrate human cells, is delivered to the nucleus and has been shown repairing UV-induced DNA damage in cell culture and human skin explants (Ceccoli et al., 1989; Yarosh et al., 1991; Gilchrest et al., 1993). Preparations with liposomes containing T4N5 have been extensively investigated in mice (Wolf et al., 1993, 1995; Yarosh et al., 1994) and have been employed in prevention studies of skin cancer in patients with the genetic disease Xeroderma pigmentosum and other individuals prone to multiple skin cancers (Wolf et al., 2000; Yarosh et al., 2001). Similarly, photolyase from Cyanobacteria and enzymes with DNA repair activity from *Micrococcus luteus* have been formulated into liposomes for use on humans’ skin (Stege, 2001; Hofer et al., 2011). Such preparations have been administered in clinical studies and helped to modulate the UV-induced immune response, as described by its experimental use in patients with polymorphic light eruption (Hofer et al., 2011) and in turn may lead to changes in microbiome colonization.

## Perspective

It is likely that UV-R leads to various changes within the landscape of microbial communities of the skin. We hypothesize that exposure to UV-R leads to alterations in skin commensals leading to change in quantity and spread of certain, defined bacteria or growth of opportunistic microorganisms. This could disrupt the equilibrium with the host immune system and trigger a local immune response. For example, when skin is colonized with *Staphylococcus aureus*, this can lead to induction of a local response by producing δ-toxin, which is involved in mast cell degranulation and thus promotes innate and adaptive type 2 response, as seen in allergic skin disease (Nakamura et al., 2013). However, on the other side it is known that UV-R can reduce the growth of *Staphylococcus aureus in vivo* and *in vitro* (Jekler et al., 1992; Yoshimura et al., 1996; Thyssen et al., 2015) and also decrease the production of super antigens, which are known to be potential triggers of immune responses (Yoshimura-Mishima et al., 1999; Thyssen et al., 2015). UV-R can also directly breakdown cell structures of microbes, leading to production of various microbial signals (**Figure 2**). They can then be recognized by the immune system and reacted against appropriately. UV-R can induce pyrimidine dimers in DNA and can have a drastic effect on the microbial communities. Furthermore, microbial DNA photoproducts could even be a potential trigger of immune responses (Rothschild, 1999). New technologies such as high-throughput sequencing and the availability of germ-free animal models, will allow us to extend our knowledge of the relationship between UV-R, the skin microbiome and immunity. One could directly study the effects of UV-R on the microbiome by taking skin swabs from the human body and directly extract DNA for further analysis but also cultivating skin microbes obtained from skin swabs and exposing them *in vitro* to different doses of UV-R in order to study the response, damage, mutations etc. This will lead to a better understanding of various photosensitive conditions, give further insights into the mechanisms of medical phototherapy and elucidate the role of the skin microbiome and its potential modulation by UV-induced immune suppression (Bouslimani et al., 2015).

## Conclusion

The skin microbiome plays an important role in developing and maintaining homeostasis and regulation of the host immune system. With increasing incidence of UV-induced skin conditions, the importance of the skin immune system in maintaining tolerance toward the resident microbiome may be crucial. Since the skin microbiome can interact with and co-control both the innate and adaptive immune system, it is of great importance to understand its relationship with the host immune system.

## Author Contributions

VP drafted the manuscript. PW and SB contributed to the draft. All authors revised and approved the final version of the manuscript.

## Conflict of Interest Statement

The authors declare that the research was conducted in the absence of any commercial or financial relationships that could be construed as a potential conflict of interest. The handling Editor declared a shared affiliation, though no other collaboration, with the authors VP and PW, and states that the process nevertheless met the standards of a fair and objective review.
